# Low Contact Resistance Organic Single‐Crystal Transistors with Band‐Like Transport Based on 2,6‐Bis‐Phenylethynyl‐Anthracene

**DOI:** 10.1002/advs.202400112

**Published:** 2024-03-18

**Authors:** Yanan Sun, Xiaosong Shi, Yamin Yu, Zhilei Zhang, Miao Wu, Limei Rao, Yicai Dong, Jing Zhang, Ye Zou, Shengyong You, Jie Liu, Ming Lei, Chuan Liu, Lang Jiang

**Affiliations:** ^1^ Beijing National Laboratory for Molecular Sciences Key Laboratory of Organic Solids Institute of Chemistry Chinese Academy of Sciences Beijing 100190 China; ^2^ University of the Chinese Academy of Sciences Beijing 100049 China; ^3^ State Key Laboratory of Information Photonics and Optical Communications and School of Science Beijing University of Posts and Telecommunications Beijing 100876 China; ^4^ Institute of Applied Chemistry Jiangxi Academy of Sciences Nanchang 330096 China; ^5^ State Key Laboratory of Optoelectronic Materials and Technologies Guangdong Province Key Laboratory of Display Material and Technology School of Physics and Engineering School of Microelectronics Sun Yat‐sen University Guangzhou 510275 China

**Keywords:** contact resistance, organic field‐effect transistors, single‐crystal

## Abstract

Contact resistance has become one of the main bottlenecks that hinder further improvement of mobility and integration density of organic field‐effect transistors (OFETs). Much progress has been made in reducing contact resistance by modifying the electrode/semiconductor interface and decreasing the crystal thickness, however, the development of new organic semiconductor materials with low contact resistance still faces many challenges. Here, 2,6‐bis‐phenylethynyl‐anthracene (BPEA) is found, which is a material that combines high mobility with low contact resistance. Single‐crystal BEPA OFETs with a thickness of ≈20 nm demonstrated high mobility of 4.52 cm^2^ V^−1^ s^−1^, contact resistance as low as 335 Ω cm, and band‐like charge transport behavior. The calculated compatibility of the *E*_HOMO_ of BPEA with the work function of the Au electrode, and the decreased |*E*_HOMO_‐***Φ***_Au_| with the increase of external electric field intensity from source to gate both contributed to the efficient charge injection and small contact resistance. More intriguingly, *p*‐type BPEA as a buffer layer can effectively reduce the contact resistance, improve the mobility, and meanwhile inhibit the double‐slope electrical behavior of *p*‐channel 2,6‐diphenyl anthracene (DPA) single‐crystal OFETs.

## Graphical abstract

OFETs based on BPEA single crystals are fabricated with an ultralow contact resistance of 335 Ω cm and band‐like transport. In addition, the introduction of BPEA thin films (6 nm) can greatly improve the charge injection and reduce the contact resistance of DPA single crystal devices despite the increase of the functional layer thickness, and thus improve the mobility.

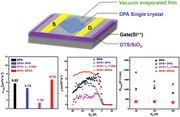

## 1 Introduction

Organic semiconductor single crystals, due to their absence of grain boundaries and highly ordered molecular arrangements, can be used as an ideal platform for fabricating high‐mobility organic field‐effect transistors (OFETs) and investigating the intrinsic properties of organic semiconductor materials.^[^ ^1^, ^2^, ^3^, ^4^, ^5^, ^6^, ^7^ ^]^ However, there are still many factors that impede the further improvement of device performance, such as high contact resistance (*R*_c_).^[^ ^8^, ^9^, ^10^ ^]^ Contact resistance not only hinders the effective injection of charge carriers, causing a voltage drop from the source/drain electrode to the conductive channel, affecting the mobility of the devices but also causing high energy consumption, hindering further miniaturization and integration of the devices. In OFETs, the *R*_c_ of the device includes two parts: the interface injection resistance (*R*_int_) between the metal/semiconductor and the access resistance (*R*_acc_).^[^ ^11^, ^12^ ^]^ *R*_int_ is caused by non‐ohmic contact due to the mismatch between the metal work function and the frontier energy levels of semiconductors. Up to now, a series of methods have been developed to reduce *R*_int_, including inserting buffer layers such as MoO_3_^[^ ^13^, ^14^ ^]^ and Mo(tfd)_3_^[^ ^15^ ^]^ to improve charge injection, modifying self‐assembled monolayers (SAMs)^[^ ^16^ ^]^ to adjust the work function of metal electrodes, etc. *R*_acc_ originating from the bulk thickness of semiconductors can be effectively relieved by reducing the crystal thickness,^[^ ^17^ ^]^ adjusting the organic thin film thickness,^[^ ^18^ ^]^ doping,^[^ ^19^ ^]^ and constructing the bottom contact device.^[^ ^20^ ^]^ Although a large number of methods have been explored to reduce the contact resistance of devices,^[^ ^21^ ^]^ organic semiconductor bulk crystals with intriguingly low *R*_c_ have been rarely reported.

The active organic semiconductor materials are the key component influencing the performance of the OFETs. Small‐molecule semiconductors play important roles in transistors because of their high purity and ordered packing compared with polymers, and the structure‐charge‐transport property relationships have been gradually recognized.^[^ ^22^ ^]^ As typical small molecule semiconductors, anthracene derivatives with extended conjugated structures are widely used as the active layer in OFETs,^[^ ^23^, ^24^, ^25^, ^26^, ^27^, ^28^, ^29^ ^]^ such as the star material 2,6‐diphenyl anthracene (DPA).^[^ ^23^, ^24^ ^]^ However, DPA suffers severe *R*_c_ which results in the double‐slope electrical behavior.^[^ ^24^ ^]^ It has been demonstrated that materials with larger π‐conjugation would possess shallower highest occupied molecular orbital (HOMO) levels, which stimulates our study in 2,6‐bis‐phenylethynyl‐anthracene (BPEA). Since BPEA possesses a similar structure to DPA but with a longer π‐conjugation length, high mobility, and low contact resistance might be anticipated in BPEA single crystals.

In this work, we demonstrate that BEPA is a material with high mobility, low contact resistance, and band‐like transport behavior. Mobility up to 4.52 cm^2^ V^−1^ s^−1^ and *R*_c_ as low as 335 Ω cm was achieved for ≈20 nm thick BPEA singlecrystal OFETs. For the merits of the high‐quality single crystals, neat interface, and low contact resistance of BPEA OFETs, band‐lile charge transport was achieved. To alleviate the *R*_c_ in DPA singlecrystal OFETs, coupled with the favorable low *R*_c_ of the BPEA device, we introduced 6 nm BPEA into the DPA single crystal device as a buffer layer, and successfully reduced the *R*_c_ of the DPA device to 35% of the original value and increased the average mobility by 15.8%. By inserting BPEA thin films (6 nm) between DPA single crystals and gold electrodes, the double‐slope non‐ideal electrical behavior was effectively inhibited in the DPA single‐crystal devices.

## 2 Result and Discussion

**Figure 1:**
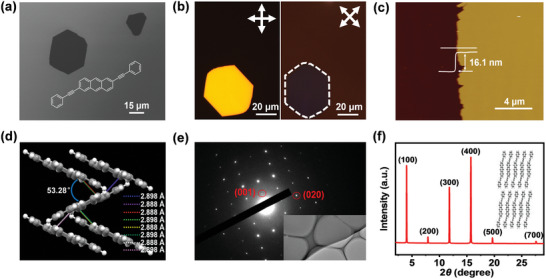
a) SEM image b) polarized optical image and c) AFM image of BPEA single crystals. d) Crystal packing of BPEA in bc‐plane. e) TEM image and its corresponding SAED pattern. f) XRD patterns of BPEA single crystals grown on OTS‐treated SiO_2_/Si substrate.

Single crystals of BPEA were grown by the physical vapor transport (PVT) method using a horizontal tube furnace. A quartz boat containing BPEA powder was placed in a high‐temperature zone (195 °C) with pure argon gas as a carrier. Single crystals of BPEA were obtained on the octadecyltrichlorosilane (OTS) modified SiO_2_ (300 nm)/Si substrate placed in a low‐temperature zone (80–100 °C). **Figure** **1**a shows the scanning electron microscopy (SEM) image of the obtained single crystals with a typical size of tens of micrometers. The homogeneous color of the polarized optical image shown in Figure 1b indicated the uniformity of the individual crystal. The color of the crystal changed from bright to dark when it was rotated from 0° to 45°, suggesting its single‐crystal nature. The atomic force microscopy (AFM) image shown in Figure 1c demonstrated the flat surface of the single crystal, which is important for the formation of intimate contact between the crystal and the electrode during device fabrication. AFM was also used to estimate the thickness of the obtained crystals with results of several nanometers to tens of nanometers shown in Figure S1, (Supporting Information).

To investigate the crystallographic structure, large‐size BPEA single crystals were prepared by the PVT method under ambient pressure for single‐crystal X‐ray diffraction (XRD) measurement (Crystal data and structure refinement shown in Table S1, Supporting Information). The results revealed that crystals of BPEA belong to the P2(1)/c space group with crystal parameters of a = 22.729(8) Å, b = 5.789(9) Å, c = 7.465(8) Å and β = 91.483(3)°. From the crystal‐structure analysis, molecules of BPEA are determined to be packed in a herringbone geometry in crystals with herringbone angle of ≈53.28° (Figure 1d). Besides, multiple C─H─π interactions (distance: ≈2.88 Å) were observed between every molecule and its neighboring four molecules. For the micro‐nano crystals, transmission electron microscopy (TEM) image of an individual crystal and its corresponding selected area electron diffraction (SAED) pattern are shown in Figure 1e. The SAED pattern could be indexed according to the lattice parameters obtained from bulk crystal. The XRD pattern of crystals obtained on OTS‐modified SiO_2_ (300 nm)/Si substrate by PVT method is shown in Figure 1f. The baseline is very straight and the peaks are very sharp, indicating that crystals of high quality are produced. The peaks in this pattern can be indexed to be (h00) according to the single crystal structure. The primary (100) peak showed strong diffraction with a d‐spacing value of 22.69 Å which was very close to the length of BPEA (22.77 Å). Besides, step‐like structures were observed for single crystals grown in the low‐temperature zone and the average height of steps was measured to be ≈22.64 Å (shown in Figure S2, Supporting Information), which was in good agreement with the d‐spacing value deduced from XRD analysis, confirming the layer‐by‐layer growth of BPEA single crystals.

**Figure 2:**
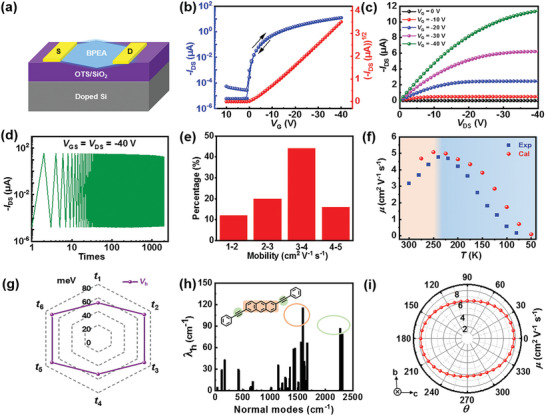
a) Schematic diagram of the BPEA single‐crystal OFET device. b) Typical transfer and c) output characteristics of BPEA single‐crystal OFETs. d) Continuous electrical test, *V*_DS_ was −40 V, with *V*_G_ switching from 0 to −40 V. e) The mobility distribution of the devices. f) Temperature dependence of mobility of experimentally measured and theoretically calculated. g) Hole transfer integral for transport paths. h) Normal modes decomposition of hole recombination energy. i) Anisotropic mobility of BPEA.

Transistors based on BPEA single crystals were fabricated using the gold‐film transferring method with bottom‐gate top‐contact configuration. The schematic of the device is shown in **Figure** **2**a. Typical transfer and output characteristics of devices based on individual single crystals are shown in Figure 2b,c. The devices demonstrated weak hysteresis, which implied that the interface between the in situ grown crystal and the OTS‐modified SiO_2_ was of high quality with limited charge‐trapping effects. The stability of the transistors based on BPEA single crystals was also studied. As shown in Figure 2d and Figure S3a,b (Supporting Information), the single‐crystal‐based transistors exhibit high stability in cycle tests and under ambient storage. The saturation mobility distribution based on 30 devices is shown in Figure 2e. More than 80% of the devices exhibited mobility above 2 cm^2 ^V^−1 ^s^−1^ with the highest value of 4.52 cm^2 ^V^−1 ^s^−1^, which is ≈10 times the mobility of thin‐film devices.^[^ ^29^ ^]^ Furthermore, the temperature‐dependent mobility study was conducted on BPEA single‐crystal OFETs. The mobility temperature relationship measured by the experiment is shown in the blue box in Figure 2f. The results show that an increase of mobility was observed from 3.18 to 4.76 cm^2^ V^−1^ s^−1^ with temperature decreasing from 300 to 240 K, indicating band‐like transport in this temperature range.^[^ ^30^, ^31^, ^32^, ^33^, ^34^ ^]^ The mobility decreased from 4.76 to 0.17 cm^2^ V^−1^ s^−1^ when further decreasing the temperature from 240 to 80 K, demonstrating the thermally‐activated behavior of the charge carriers, which was dominated by shallow traps.^[^ ^35^, ^36^ ^]^ In this case, the mobility (*μ*) follows the relationship:

(1) $$\mu \;=\mu_{0}\;\exp\left(-\frac{E_{A}}{k_{B}T}\right)$$

where *E*_A_ is the activation energy, *k*_B_ is the Boltzmann constant, *T* is the temperature, and a value of 35.9 meV for *E*_A_ can be calculated as shown in Figure S3c, (Supporting Information).

It is known that the charge transport properties of organic semiconductors are highly sensitive to the relative orientations and packing characteristics of the organic molecules. The charge delocalization in molecular crystals is dominated by the inter‐molecular charge transfer integral, while the intra‐molecular reorganization energy characterizes the trap effect arising from the electron–vibration coupling. For the charge transfer process, the charge transfer rate can be calculated from the first principle by using the hopping model described by Marcus charge transfer rate formula:^[^ ^37^ ^]^

(2) $$k\;=\frac{V^{2}}{\hbar }\;\sqrt{\frac{\pi }{\lambda k_{B}T}}\exp\left(-\frac{{\left(\lambda -\Delta G^{0}\right)}^{2}}{4\lambda k_{B}T}\right)$$

where ***k*** is the hole/electron transfer rate, ***V*** is the transfer integral between the initial and final states, ***λ*** is the reorganization energy, which is defined as the energy change associated with the geometry relaxation during the charge transfer, and **Δ*G* ^0^** is the relevant change of total Gibbs free energy. In the self‐exchange reaction, **Δ*G* ^0^** equals zero. ***k*_B_** is the Boltzmann constant, *T* is the temperature, ℏ is the reduced Planck constant.

The particle diffusion coefficient (*D*) can be obtained by random walk Monte Carlo simulation:

(3) $$D\;=\;\frac{1}{2n}\lim_{t\to \infty }\frac{\langle r^{2}\rangle }{t}$$

Finally, the mobility of materials can be predicted by Einstein formula:

(4) $$\mu \;=\;\frac{e}{k_{B}T}D$$

where *e* is the electron charge.

Based on the BPEA crystal structures, the transfer integral between central and neighboring molecules (lat_cutoff is 6 Å for neighbor list construction), reorganization energy (*λ*), and charge carrier mobility were calculated by MOMAP package at the B3LYP/6‐31G**.^[^ ^37^, ^38^, ^39^, ^40^ ^]^ According to the crystal structure of the systems and the calculated transfer integrals, we divide the studied systems into two categories: π–π stacking (*t*_1_, *t*_4_) and herringbone stacking (*t*_2‐3_, *t*_5‐6_), as shown in Figure S3d (Supporting Information). The transfer integral values of all the transport paths are shown in Figure 2g where the hole transfer integral of π–π stacking categories is −53 meV, and that of herringbone stacking categories is −71 meV. Since the recombination energy of holes (137 meV) is smaller than that of electrons (165 meV), the BPEA crystal is dominated by hole transport. The reorganization energy can be decomposed into all the vibration mode relaxations, as shown in Figure 2h. From the normal mode analysis of the recombination energy, we can see that the high‐frequency part (1350–1700 cm^−1^) has the main contribution to the recombination energy. Based on the above calculation of parameters, the predicted mobility–temperature relationship from 300 to 50 K based on Equations (2)–(4) was shown in Figure 2f, which was in good agreement with the experimental results. The results of anisotropic mobility in bc‐plane are shown in Figure 2i, indicating BPEA is a high‐mobility material with calculated *μ* over 6**_ _** cm^2^ V^−1^ s^−1^, showing anisotropic charge transport with *µ*_max_/*µ*_min_ = 7.78/6.26 = 1.24.

Compared with the nonlinear *I*_DS_‐*V*_DS_ behavior in the output curve for high‐mobility DPA OFETs,^[^ ^23^, ^24^ ^]^ linear *I*_DS_‐*V*_DS_ was observed at low *V*_DS_ for BPEA OFETs, demonstrating low contact resistance. Thus transmission‐line method (TLM) is adopted to evaluate *R*_c_ in BPEA single‐crystal OFETs. The formula for calculating contact resistance using the TLM^[^ ^41^, ^42^ ^]^ is:

(5) $$R_{\mathrm{total}}W\;=R_{c}\;W+\frac{L}{\mu C_{i}\left(V_{G}-V_{T}\right)}$$

where *R*_c_ is the contact resistance of the device, *R*_total_ is the total resistance of the device. *V*_G_, and *V*_T_ are gate‐source voltage, and threshold voltage, respectively. *C*_i_ is the unit dielectric capacitance. *W* and *L* are channel width and length, respectively.

**Figure 3:**
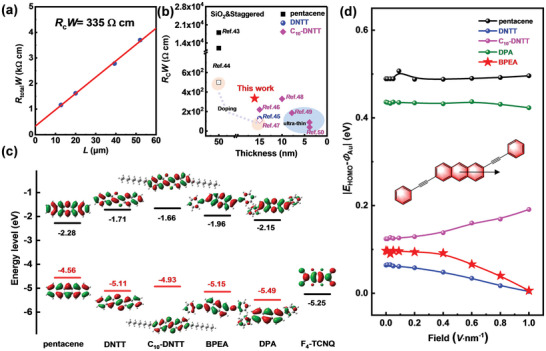
a) TLM plots of BPEA single‐crystal OFETs. b) Summarized *R*_c_ values of BGTC OFETs with *R*_c_ *W* based on SiO_2_ substrate.^[^ ^44^, ^45^, ^46^, ^47^, ^48^, ^49^, ^50^, ^51^ ^]^ The full and open symbols represent the *R*_c_ *W* obtained by the pristine and doped devices, respectively. The gray‐blue area contains the *R*_c_ *W* data from ultrathin devices. c) Frontier molecular orbitals by DFT(B3LYP/6‐31G*). d) The change of |*E*_HOMO_‐*Φ*_Au_| with simulated external electric field (0–1 *V* nm^−1^) along the molecular long axis.

In the TLM measurements, the channel width of BPEA devices was fixed at 40 µm, and the length of the channel was 52.07, 39.35, 20.00, and 12.73 µm, respectively. The transfer curves in the linear region were tested (*V*_DS_ = −2 V, *V*_G_ = −40 V). By fitting the *R*_total_ *W* versus channel length data (**Figure** **3**a) based on Equation (5), the intercept of this curve is the contact resistance (*R*_c_ *W*) of the device according to the above calculation Equation (5). The estimated width‐normalized contact resistance of 335 Ω cm could be obtained, this is much lower than the *R*_c_ values of reported high‐mobility anthracene derivatives, such as DPA and (E)‐2‐styrylanthracene (2‐phvA)^[^ ^43^ ^]^ as shown in Figure S4 (Supporting Information). The extracted *R*_c_ *W* was compared with the star semiconductors including pentacene, Dinaphtho[2,3‐b:2',3'‐f]thieno[3,2‐b]thiophene (DNTT), and 2,9‐didecyldi‐naphtho[2,3‐b:2',3'‐f]thieno[3,2‐b]thiophene (C_10_‐DNTT) as shown in Figure 3b. For devices fabricated on SiO_2_/Si substrates adopted BGTC configuration, ≈20 nm‐thick BPEA device shows comparable *R*_c_ *W* value to DNTT and C_10_‐DNTT. To this end, we calculated the energy levels of the above systems by Frontier Molecular Orbitals (FMOs) theory based on mono‐molecule at B3LYP/6‐31G** as shown in Figure 3c. And characterized the work function of Au electrodes and the BPEA film by ultra‐violet photoelectron spectroscopy (UPS) (shown in Figure S5, Supporting Information). The results showed that the HOMO energy level of BPEA was more compatible with the work function of the Au electrode, which was the main reason for its low contact resistance. On this basis, we simulated the **|*E*_HOMO_‐*Φ*_Au_|** under different external electric field from the source to the gate. As shown in Figure 3d, the **|*E*_HOMO_‐*Φ*_Au_|** of BPEA and DNTT decrease to close to 0 with the increase of external electric field intensity, which also demonstrated efficient charge injection and small contact resistance of BPEA and DNTT.

**Figure 4:**
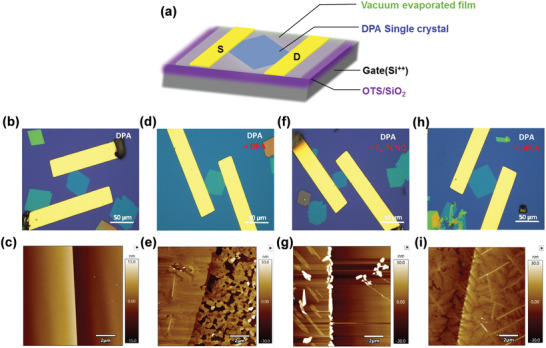
a) Schematic illustration of BGTC OFETs. Optical microscopic images and AFM images of b,c) DPA single‐crystal device; d,e) device of DPA + 6 nm DPA thin film; f,g) device of DPA + 6 nm F_4_‐TCNQ thin film; h,i) device of DPA + 6 nm BPEA thin film.

**Figure 5:**
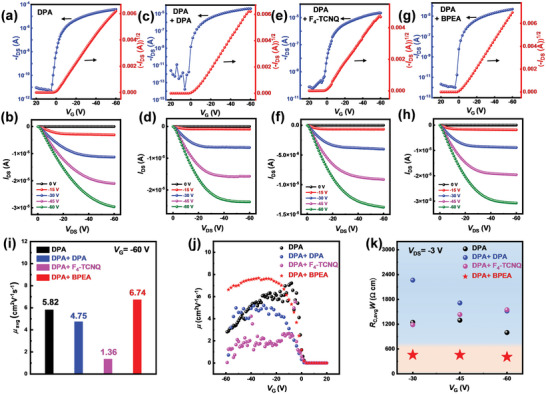
Typical transfer and output curves of a,b) DPA single crystal, c,d) DPA single crystal + 6 nm DPA film, e,f) DPA single crystal +6 nm F_4_‐TCNQ film and g,h) DPA single crystal +6 nm BPEA film. i) Average mobility of devices with *V*_G _= −60 V. j) Gate voltage‐dependent mobility for different types of devices. k) Contact resistance extracted using the G‐function.

For the merits of the low contact resistance of BPEA, we explored its possibility of alleviating the contact resistance of novel anthracene semiconductor DPA OFETs. By adopting DPA single crystals with a similar thickness of about 17 nm, we fabricated DPA single crystal OFETs. Systematical comparisons were carried out by inserting different buffer‐layer materials of the same thickness (≈6 nm), including DPA, 2,3,5,6‐tetrafluoro‐7,7,8,8‐tetracyanoquinodimethane (F_4_‐TCNQ), and BPEA to investigate their effects on the contact resistance. Schematic illustration of BGTC OFETs, optical microscopic images (scale bar, 50 µm) of devices, and AFM images of corresponding functional layers are shown in **Figure** **4**. It can be observed that compared with F_4_‐TCNQ, better coverage, and smaller roughness could be observed for 6 nm BPEA and DPA on top of DPA single crystals, which guarantees efficient electrode/semiconductor contacts. Typical transfer and output curves of corresponding devices are illustrated in **Figure** **5**a–h. The average mobility of all field‐effect devices in the same batch with *V*_G _= −60 V is shown in Figure 5i. The average mobility of DPA + 6‐nm BPEA OFETs is 6.74 cm^2 ^V^−1 ^s^−1^, which is 15.8% higher than that of DPA single‐crystal devices. Then, we extracted mobilities at different *V*_G_, as shown in Figure 5j, to demonstrate the gate‐dependent phenomenon of the device. The results show that the introduction of BPEA significantly eliminated the gate‐dependent mobility of DPA single‐crystal devices.

To obtain direct evidence of the small contact resistance, the contact resistance values were extracted by adopting the G‐function method.^[^ ^52^, ^53^ ^]^ The contact resistance is calculated through the output curve of the saturation zone, and its calculation formula is:

(6) $$\begin{array}{c} R_{c}=R_{\mathrm{total}}\;-R_{\mathrm{channel}}\\ =\frac{1}{G}\;-\frac{1}{\mathrm{intercept}\left(G,V_{D}\right)+\mathrm{slope}\left(G,V_{D}\right)*V_{\mathrm{DS}}} \end{array}$$

(7) $$G\;=\frac{I_{\mathrm{DS}}}{V_{\mathrm{DS}}}\;$$

Among them, the slope and intercept of the curve can be obtained through the G‐*V*_DS_ relationship curve based on Equations (6)–(7). The *R*_c,avg_ *W*‐*V*_DS_ curses of the same batch of devices are shown in Figure S6 (Supporting Information). The extracted contact resistance values of the devices with *V*_DS _= −3 V are shown in Figure 5k. The contact resistance of the DPA + 6 nm BPEA thin film device at gate voltage of −45 V is 455 Ω cm. This value is much smaller than that of devices of DPA single crystal with DPA and F_4_‐TCNQ thin film, while the contact resistance of DPA single crystal at a *V*_G_ of −45 V is ≈1.29 kΩ cm (Contact resistance data extracted using the G‐function shown in Table S2, Supporting Information). These results show the DPA + 6 nm BPEA devices demonstrate slightly higher mobility than pristine DPA devices, negligible gate‐dependent mobility, and the lowest contact resistance among these four types of devices. Therefore, the introduction of BPEA can greatly improve the charge injection and reduce the contact resistance of DPA single‐crystal devices despite the increase in the functional layer thickness. This might be the possible reason for the increase in device mobility as shown in Figure 5i. The effect of the 6 nm BPEA buffer layer in reducing the contact resistance was also effective in 2‐phvA single‐crystal devices as shown in Figure S7 (Supporting Information). In conclusion, BPEA is not only a material with high mobility and low contact resistance but also has good general adaptability as a buffer layer for reducing the contact resistance of OFETs with deep HOMO levels.

## 3 Conclusion

In conclusion, single crystals of BPEA were fabricated by the physical vapor transport technique. Transistors based on BPEA single crystals demonstrated high mobility up to 4.52 cm^2^ V^−1^ s^−1^ and low contact resistance with a value of 335 Ω cm, which is comparable to that of novel DNTT material‐based devices. The low contact resistance of BPEA devices could be attributed to the small mismatch between the HOMO level and the work function of gold electrodes and the decreased |*E*_HOMO_‐*Φ*_Au_| with the increase of external electric field intensity. Owing to the high‐quality crystals, neat interface, and low contact resistance of BPEA OFETs, band‐like charge transport behavior was achieved both in experimental and theoretical calculations. In addition to that, by inserting BPEA thin films (6 nm) between DPA single crystals and gold electrodes, the contact resistance of DPA single crystal devices was significantly reduced (down to 35% of its pristine value), which effectively inhibits the double‐slope non‐ideal electrical behavior, rendering possible application of BPEA in high‐performance OFETs.

## 4 Experimental Section

### Materials

BPEA was synthesized following the procedure reported in the literature.^[^ ^29^ ^]^ Phenylacetylene, trans‐dichlorobis(triphenyl phosphine)palladium were purchased from Sigma Aldrich and were used without further purification. Tetrahydrofuran (THF) and diethylether were dried over sodium/benzophenone. SiO_2_ (300 nm)/Si substrates used for BPEA single crystal growth and transistor fabrication in this study were cleaned with deionized water, pure isopropanol, piranha solution (H_2_SO_4_/H_2_O_2 _= 7:3), deionized water and pure isopropanol successively and then blow–dried under nitrogen. The substrates were further treated with O_2_ plasma for 5 min. The substrates were then placed in a vacuum‐pumped oven at 90 °C for 1 h to eliminate the moisture. For the following surface modification, a droplet of octadecyltrichlorosilane (OTS) was added and the oven was kept vacuum pumped at 120 °C for 2 h. Finally, the substrates were cleaned with hexane, chloroform, isopropanol and then blow‐dried under nitrogen. The single crystals of BPEA were synthesized on the OTS‐modified substrates by the physical vapor transport technique using a two‐zone horizontal‐tube furnace evacuated by a mechanical pump which kept the pressure at 20 Pa. A quartz boat containing BPEA powder was placed in the high‐temperature zone (195 °C) with high‐pure argon gas as carrier (50 sccm). Single crystals of BPEA were obtained on the substrates in the low‐temperature zone from 80 to 100 °C. Transistors with bottom‐gate top‐contact configuration were fabricated by gold‐film transferring method based on the OTS‐modified SiO_2_ (300 nm)/Si substrate.

### Characterization

The optical microscopy images were taken with Leica DM4 M. Transmission electronic microscopy (TEM) images and selected area electron diffraction (SEAD) was conducted on a JEOL JEM‐1011. X‐ray diffraction (XRD) for investigating the crystallinity of BPEA crystals was carried out using a D/max2500 with monochromatic Cu Kα radiation. Atomic force microscopy (AFM) images were obtained using a Cypher Asylum Research with tapping mode. Scanning electron microscopy (SEM) images were obtained using a Hitachi S‐4800. The OFETs were characterized using Agilent B1500 with the assistance of a probe station system in ambient conditions. The field‐effect mobility was calculated in the saturation regime. The equation used was as follows:

(8) $$I_{\mathrm{DS}}=\frac{\mu C_{i}W}{2L}\;{\left(V_{G}-V_{T}\right)}^{2}$$

where *I*_DS_, *V*_G_, and *V*_T_ are drain‐source current, gate‐source voltage, and threshold voltage, respectively. *C*_i_ is the unit dielectric capacitance. *W* and *L* are channel width and length, respectively. The transmission‐line method (TLM) was used for the estimation of width‐normalized contact resistance. The *R*_c_ could be estimated by the extraction of the intercept of the function of the width‐normalized total device resistance (*R*_total_ *W*) versus the channel length. The *R*_total_ was the ratio of *V*_DS_ to *I*_DS_ at *V*_DS _= −2 V.

### Theoretical and Computational

The frontier molecular orbital distributions of pentacene, DPA, BPEA, and other molecular structures was calculated by using first principles (B3LYP/6‐31G*). Based on the crystal structures, reorganization energy (*λ*), transfer integral (*V*) values between central and neighboring molecules, and charge carrier mobility were calculated by MOMAP package at the B3LYP/6‐31G**.

[CCDC 2323347 contains the supplementary crystallographic data for this paper. These data can be obtained free of charge from The Cambridge Crystallographic Data Centre via www.ccdc.cam.ac.uk/data_request/cif.]

## Conflict of Interest

The authors declare no conflict of interest.

## Supporting information

Supporting Information

## Data Availability

The data that support the findings of this study are available from the corresponding author upon reasonable request.
