# Supplementary material for: Low Contact Resistance Organic Single‐Crystal Transistors with Band‐Like Transport Based on 2,6‐Bis‐Phenylethynyl‐Anthracene
Source: Adv Sci (Weinh). 2024 Mar 18;11(22):2400112. doi: 10.1002/advs.202400112 (PMC11165518; doi:10.1002/advs.202400112)
Supplement: Supplementary file 1 — Supporting Information [file ADVS-11-2400112-s001.pdf]

ADVANCED SCIENCE
Open Access

Supporting Information

for *Adv. Sci.*, DOI 10.1002/advs.202400112

Low Contact Resistance Organic Single-Crystal Transistors with Band-Like Transport Based on 2,6-Bis-Phenylethynyl-Anthracene

Yanan Sun, Xiaosong Shi, Yamin Yu, Zhilei Zhang, Miao Wu, Limei Rao, Yicai Dong, Jing Zhang, Ye Zou, Shengyong You, Jie Liu, Ming Lei, Chuan Liu and Lang Jiang**

Supporting Information

Low contact resistance organic single-crystal transistors with band-like transport based on 2,6-bis-phenylethynyl-anthracene

Yanan Sun, Xiaosong Shi, Yamin Yu, Zhilei Zhang, Miao Wu, Limei Rao, Yicai Dong, Jing Zhang, Ye Zou, Shengyong You, Jie Liu, Ming Lei, Chuan Liu and Lang Jiang**

Y.-N. Sun, X.-S. Shi, Y.-M. Yu, Z.-L. Zhang, M. Wu, L.-M. Rao, Y.-C. Dong, J. Zhang, Prof. Y. Zou, Prof. J. Liu, Prof. L. Jiang
Beijing National Laboratory for Molecular Sciences, Key Laboratory of Organic Solids, Institute of Chemistry, Chinese Academy of Sciences, Beijing 100190, China.
Y.-N. Sun, X.-S. Shi, Y.-M. Z.-L. Zhang, M. Wu, L.-M. Rao, Prof. L. Jiang
University of the Chinese Academy of Sciences, Beijing 100049, China.
E-mail: ljiang@iccas.ac.cn, liujie2009@iccas.ac.cn

Prof. S.-Y. You
Institute of Applied Chemistry, Jiangxi Academy of Sciences, Nanchang 330096, China
Y.-M. Yu, Prof. M. Lei
State Key Laboratory of Information Photonics and Optical Communications and School of Science, Beijing University of Posts and Telecommunications, Beijing 100876, China.
Prof. C. Liu
State Key Laboratory of Optoelectronic Materials and Technologies, Guangdong Province Key Laboratory of Display Material and Technology, School of Physics and Engineering, School of Microelectronics, Sun Yat-sen University, Guangzhou 510275, China.

Table S1. Crystal data and structure refinement for BPEA

Empirical formula	$C_{30}H_{18}$
Formula weight	378.44
Temperature/K	169.99(10)
Crystal system	monoclinic
Space group	P21/C
a/Å	22.7298(9)

b/Å	5.7899(2)
c/Å	7.4658(2)
α/°	90
β/°	91.483(3)
γ/°	90
Volume/$Å^3$	982.20(6)
Z	2
ρ_{calc}g/cm^3	1.280
μ/mm^{-1}	0.552
F(000)	396.0
Crystal size/mm^3	0.2 × 0.1 × 0.1
Radiation	CuKα (λ = 1.54184)
2Θ range for data collection/°	7.782 to 151.292
Index ranges	-26 ≤ h ≤ 28, -4 ≤ k ≤ 6, -9 ≤ l ≤ 9
Reflections collected	5735
Independent reflections	1938 [R_{int} = 0.0306, R_{sigma} = 0.0329]
Data/restraints/parameters	1938/0/136
Goodness-of-fit on F^2	1.080
Final R indexes [I>= 2σ (I)]	R_1 = 0.0492, wR_2 = 0.1313
Final R indexes [all data]	R_1 = 0.0714, wR_2 = 0.1453
Largest diff. peak/hole / e $Å^{-3}$	0.24/-0.18

Table S2. Contact resistance extracted using the G-function.

R_cW (Ω cm) / V_G (V)	DPA	DPA + DPA	DPA+ F_4-TCNQ	DPA + BPEA
-30	1244	2265	1185	456
-45	1294	1713	1433	455
-60	998	1519	1548	408

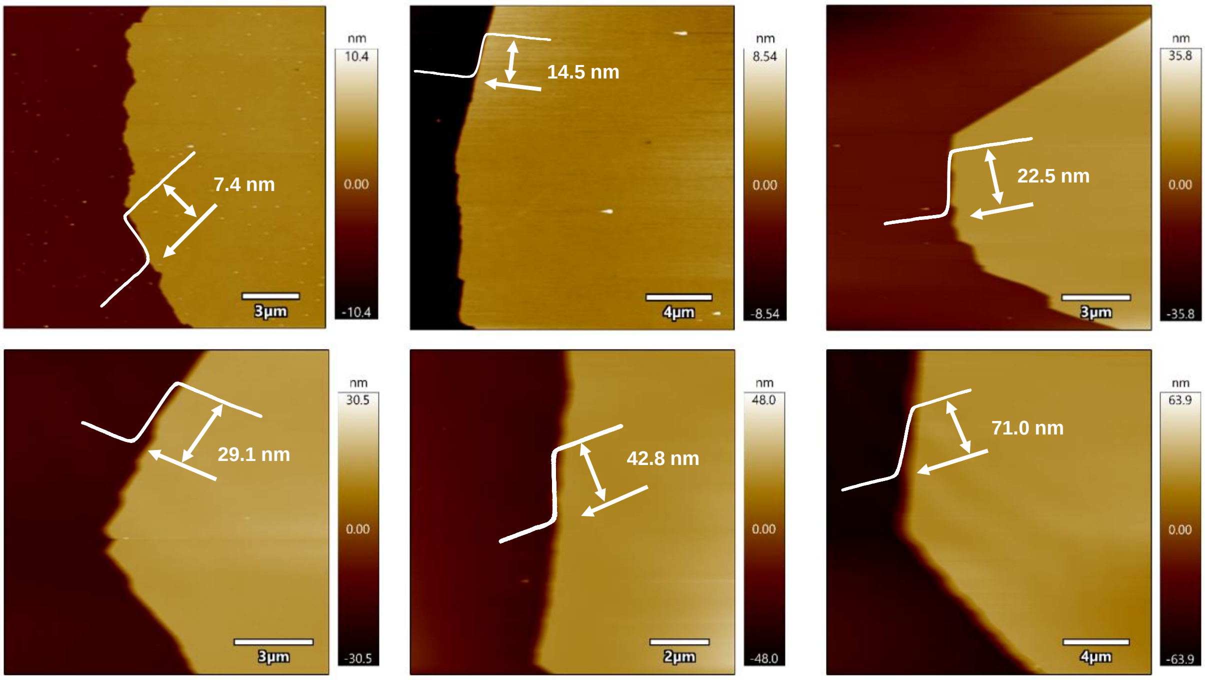

Figure S1. Crystal thickness measured by AFM ranged from several nanometers to tens of nanometers.

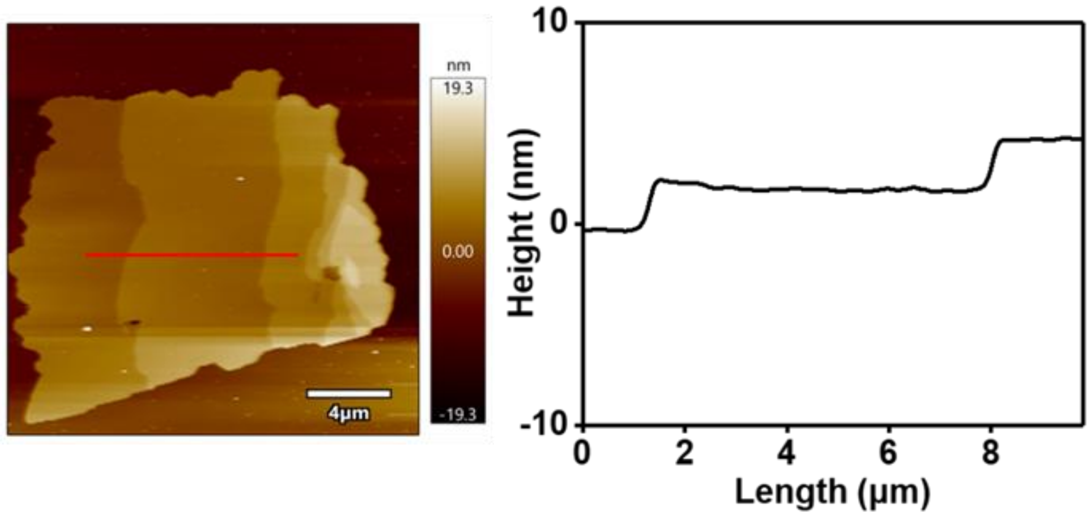

Figure S2. A step-like structure was clearly observed on the surface of single crystals grown in low-temperature zone and the average height of steps was about 2.26 nm.

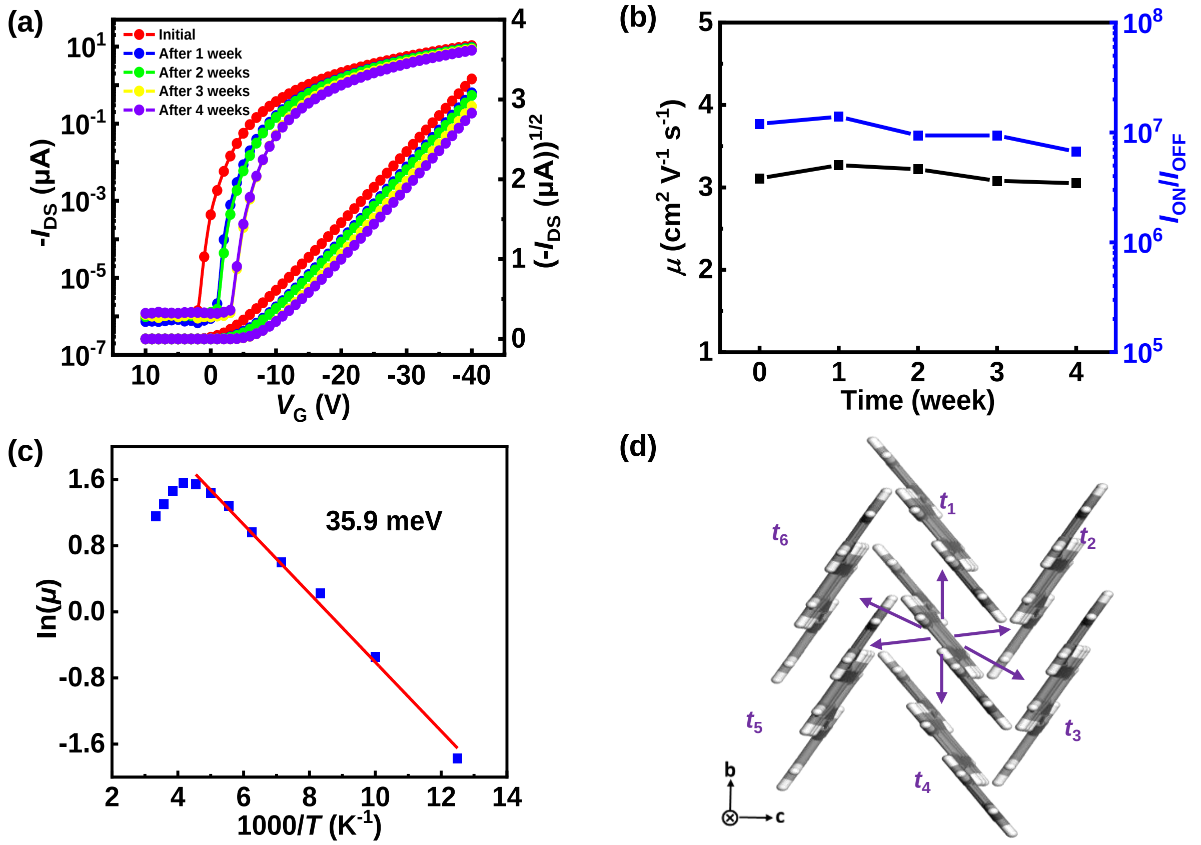

Figure S3. (a) The characteristic of stability of the transistor based on BPEA single crystal; (b) Time dependence of mobility and on/off ratio; (c) Ln (μ) versus 1000/T; (d) Transport paths from the central molecule to the neighboring molecules.

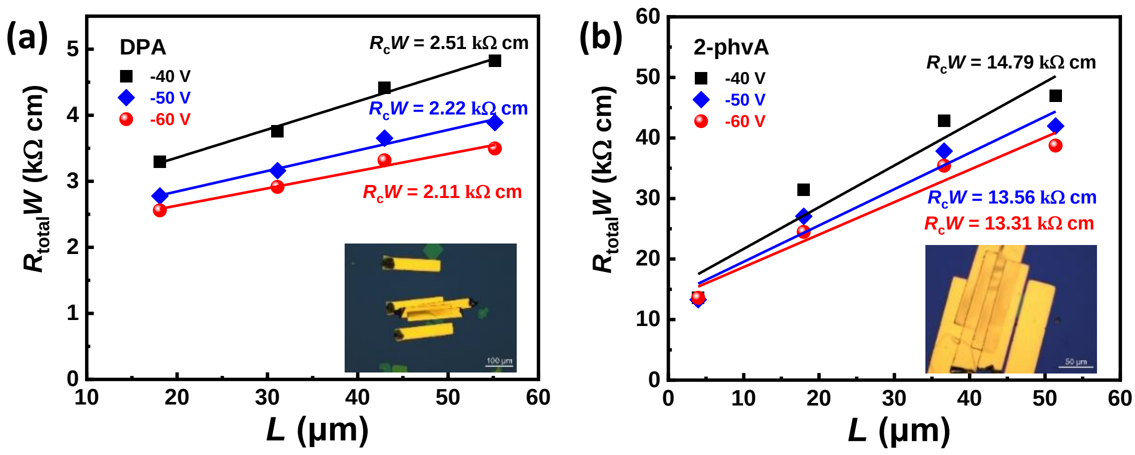

Figure S4. (a) TLM plots of DPA single-crystal OFETs with a thickness of ~17nm. (b) TLM plots of 2-phvA single crystal OFETs with a thickness of ~35nm.

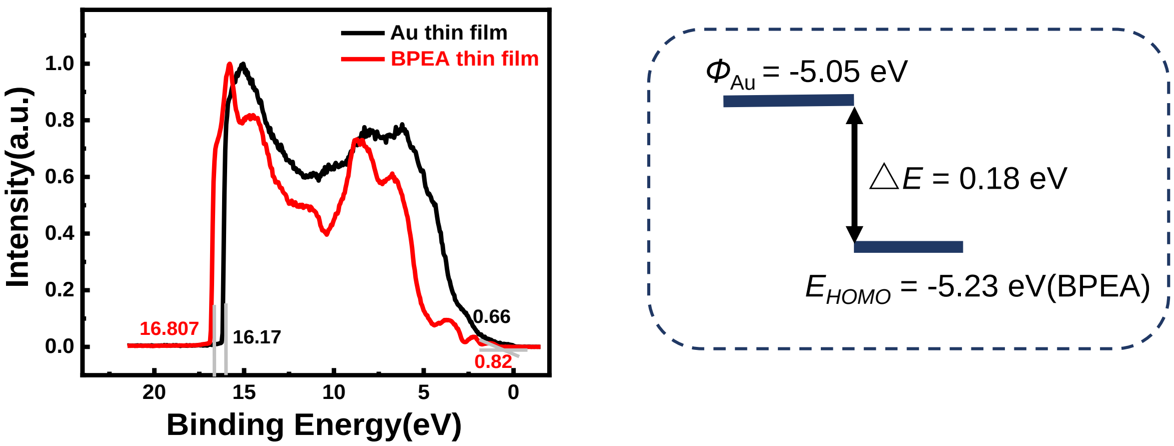

Figure S5. UPS spectrums of Au and BPEA evaporated thin films.

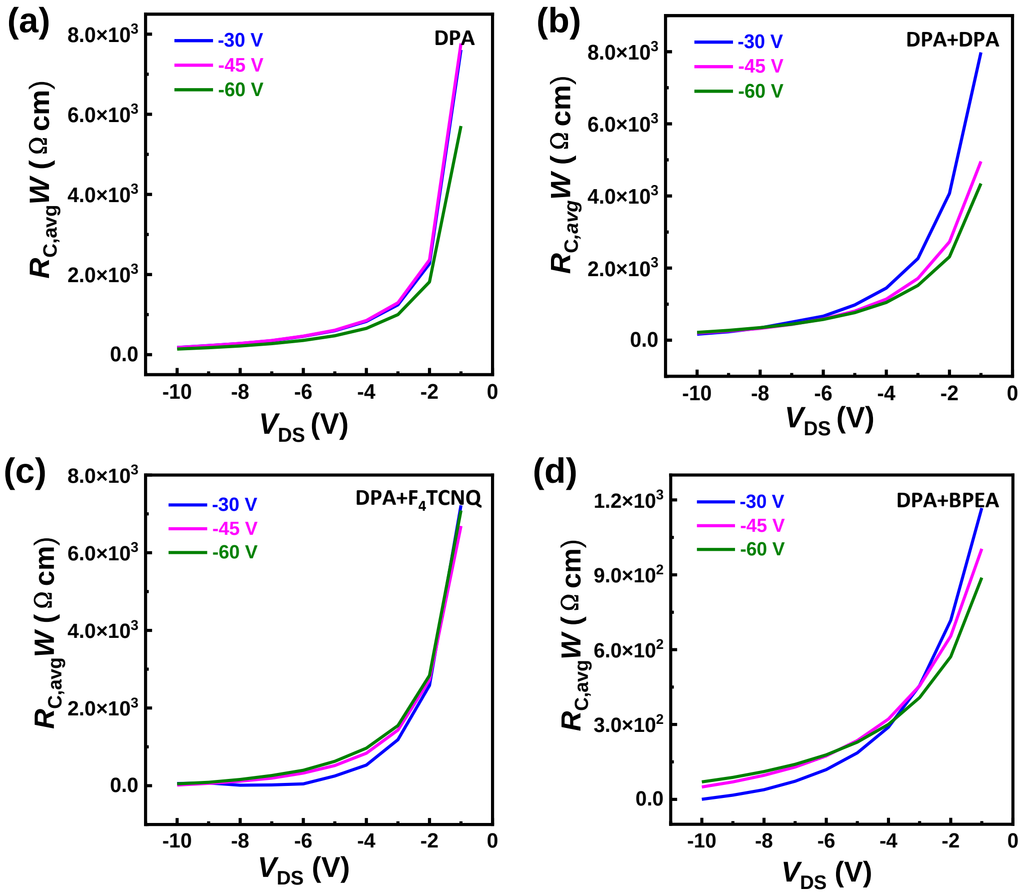

Figure S6. Contact resistance data extracted using the G-function.

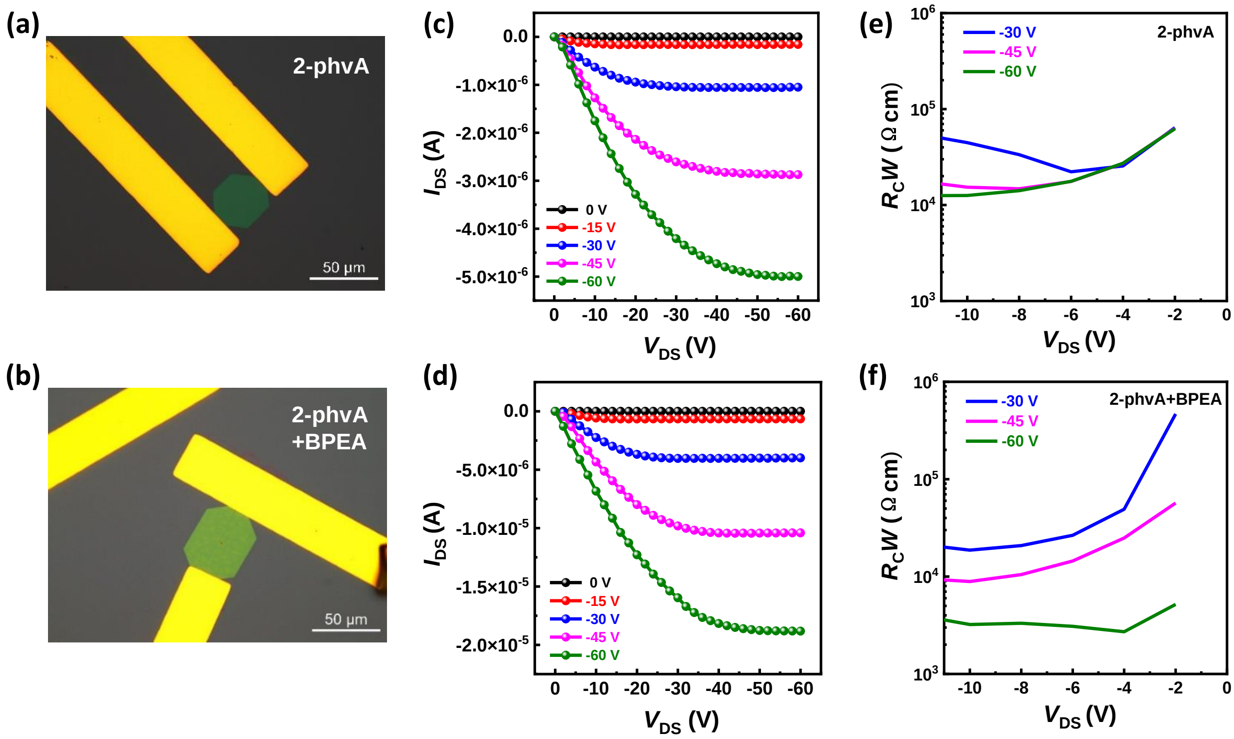

Figure S7. (a,b) Optical microscopy images of 2-phvA single crystal and 2-phvA single-crystal +6-nm BPEA thin film. (c,d) Output curves of 2-phvA single crystal and 2-phvA single-crystal +6-nm BPEA thin film. (e,f) Contact resistance extracted using the G-function.
